# Cancer‐associated fibroblasts promote M2 polarization of macrophages in pancreatic ductal adenocarcinoma

**DOI:** 10.1002/cam4.993

**Published:** 2017-01-18

**Authors:** Aibin Zhang, Yigang Qian, Zhou Ye, Haiyong Chen, Haiyang Xie, Lin Zhou, Yan Shen, Shusen Zheng

**Affiliations:** ^1^ Division of Hepatobiliary and Pancreatic SurgeryDepartment of SurgeryFirst Affiliated HospitalSchool of MedicineZhejiang UniversityHangzhouChina; ^2^ Key Laboratory of Combined Multi‐Organ TransplantationMinistry of Public HealthHangzhouChina; ^3^ Key Laboratory of Organ TransplantationHangzhouChina; ^4^ Collaborative innovation center for Diagnosis treatment of infectious diseasesHangzhouChina

**Keywords:** Cancer‐associated fibroblasts, Macrophage colony‐stimulating factor (M‐CSF), pancreatic adenocarcinoma, reactive oxygen species, tumor‐associated macrophages

## Abstract

Pancreatic ductal adenocarcinoma (PDAC) is characterized by remarkable desmoplasia with infiltration of distinct cellular components. Cancer‐associated fibroblasts (CAFs) has been shown to be among the most prominent cells and played a significant role in shaping the tumor microenvironment by interacting with other type of cells. Here, we aimed to investigate the effect of CAFs in modulating phenotype of tumor‐associated macrophages (TAM). Under treatment of CAFs conditioned medium (CM) or direct co‐culture with CAFs, monocytes exhibited enhanced expression of CD206 and CD163 compared with control group (*P *<* *0.01). The induction of M2 polarization was mediated by increased reactive oxygen species (ROS) production in monocytes as ROS elimination abolished the effect of CAFs (*P *<* *0.05). The supernatant analysis showed that pancreatic CAFs produced increased macrophage colony‐stimulating factor (M‐CSF). Upon treatment of M‐CSF neutralizing antibody, the ROS generation and M2 polarization of CAFs CM‐stimulated monocytes were significantly inhibited (*P *<* *0.05). In addition, the CAFs‐induced M2 macrophages significantly enhanced pancreatic tumor cell growth, migration, and invasion. Collectively, our data revealed that pancreatic CAFs were able to induce a tumor‐promoting TAM phenotype partly through secreted M‐CSF and enhanced ROS production in monocytes, indicating possible treatment strategies by targeting stromal cell interaction within PDAC microenvironment.

## Introduction

Pancreatic ductal adenocarcinoma (PDAC) is among the most lethal of malignancies with a median survival of 4–6 months 1. The 5‐year survival rate is only 24% even when curative surgery is performed. PDAC is uniquely featured by a striking desmoplastic reaction caused by proliferation of activated pancreatic stellate cells, which is equivalent to cancer‐associated fibroblasts (CAFs) in other type of cancers 2. It has been revealed that CAFs in PDAC is tightly associated with tumor progression through direct interaction with cancer cells, like promoting tumor cell survival, growth, and invasion as well 3, 4.

Aside from CAFs, the abundant immune cells’ infiltration also characterizes the tumor microenvironment, and further complicates the stroma 5, 6. Recent studies suggested that CAFs played an important role in interacting with other tumor‐infiltrating immune cells and modulating immunologic responses 7. There is evidence that CAFs help the pancreatic cancer cells escaping from immune surveillance 8, but the complex interfaces between CAFs and other stromal cells remain to be defined. Among the intra‐tumor immune cell population, monocytes or macrophages constitute a critical component. In PDAC, large amount of monocytes in bone marrow, peripheral blood, and spleen were recruited into tumor. Upon arriving, these cells were transformed toward distinct functional status induced by local cytokines and other signals within microenvironment, among which M1 and M2 macrophage are well characterized 9. While M1 is mainly responsible for the T_H_1 cell response and secrete pro‐inflammatory cytokines, M2 exhibits low efficiency in antigen presentation and produce high amount of anti‐inflammatory cytokines. It has been shown that M1 macrophage enhances inflammation and involves in early tumor development, whereas M2 is associated with tumor progression by promoting angiogenesis and immune suppression 10, 11, 12.

Although the phenotype modulation of macrophages was intensely studied, the impact of CAFs on this transition has not been delineated. To this end, we sought to interrogate the role of CAFs on tumor‐associated macrophages (TAM) reprogramming in PDAC.

## Material and Methods

### Antibodies and reagents

Primary antibodies for flow cytometry, immunofluorescence, immunohistochemistry, and immunoblotting include CD206 (Biolegend, 321104), CD163 (Biolegend, 333606), *α*‐SMA (Abcam, ab5694), and GAPDH (Cell Signaling, 5174). The magnetic bead for monocytes sorting is CD14 microbeads (Miltenyi Biotec, 130‐050‐201). Butylated hydroxyanisole (BHA) was obtained from Sigma.

### Primary cell isolation and culture

Briefly, fresh tumor specimen was harvested and washed a couple of times with Hank's Balanced Salt Solution (HBSS) before further handling. The tissues were then mechanically dissociated by scissors and minced with scalpel blade to obtain 1–2 mm^3^ pieces. The pieces were further incubated in RPMI 1640 medium containing collagenase IV of 200 U/mL (Worthington biochemical) and 0.1% DNase (Sigma) at 37°C under constant shaking. After enzymatic digestion, cells were filtered through 70‐ and 40 *μ*m nylon mesh to yield single cell suspension. The harvested cells were resuspended in Iscove's Modified Dulbecco's Medium (IMDM, Gibco) containing 10% fetal bovine serum (FBS, Gibco), 4 mmol/L glutamine, and 100 U/mL penicillin and 100 U/mL streptomycin, and cultured in 6‐well plates until pancreatic CAFs emerged.

Human peripheral blood mononuclear cells (PBMC) were obtained via density gradient centrifugation, and monocytes were further isolated using magnet‐mediated separation by CD14 magnetic beads. The harvested monocytes were cultured in RPMI‐1640 medium (Gibco) supplemented with 10% FBS, 100 U/mL penicillin, and 100 U/mL streptomycin. The human peripheral blood was obtained from healthy volunteers.

All studies regarding human samples were approved by Ethics Committee of the First Affiliated Hospital, Zhejiang University School of Medicine. Informed written consent was obtained from patients and volunteers according to the principles expressed in the Declaration of Helsinki.

### Cell lines

Human pancreatic cancer cell line Panc1 and Miapaca2 were purchased from the Shanghai Institution for Biological Science (Shanghai, China). These cell line were cultured with RPMI‐1640 or Dulbecco's Minimal Essential Medium supplemented with 10% fetal bovine serum, 100 U/mL penicillin, and 100 *μ*g/mL streptomycin. We have had all the cell line authenticated by a professional biotechnology company in 2015.

### Cell co‐culture

Transwell chambers (Corning, NY) containing 6.5 mm‐diameter polycarbonate filters (1 *μ*m pore) were used for cell co‐culture assay. CAFs were seeded on the lower compartments, while monocytes were cultured on the upper compartments. Monocytes were collected 2 days after co‐culture.

### CAFs conditioned medium stimulation

Pancreatic CAFs were cultured in serum‐free IMDM for 24 h, and then the supernatants were harvested as conditioned medium (CM). The primary monocytes were treated by CM for indicated times.

### Flow cytometry

Monocytes with different treatments were washed twice in PBS, and then stained with FITC‐CD206 or Phycoerythrin‐CD163 antibody for 30 min at 4°C using the recommended dilution from manufacturers. After incubation, the cells were washed twice again for examination and FlowJo software was used for analysis.

### ELISA assay

A total of 1 × 10^6^ CAFs or skin fibroblasts were seeded onto 6‐well plates in serum‐free IMDM medium, and 36 h later the supernatants were collected. The determination of cytokines concentration including macrophage colony‐stimulating factor (M‐CSF) was performed using ELIAS assays (R&D Systems) under the manufacturer's protocol.

### Determination of ROS levels

Collected monocytes were washed with PBS and then were resuspended with 10 *μ*mol/L DCFH‐DA (molecular probes) in PBS. After 30 min incubation at 37°C, cells were washed for three times with PBS. Detection of reactive oxygen species (ROS) levels was carried out via flow cytometry.

### Cell growth assay

Panc1 cells were seeded onto 96‐well plates, and received stimulation of normal monocytes medium or medium from CAFs‐stimulated monocytes. After 48 h, the cell viability was measured by Cell Counting Kit‐8 following the manufacturer's instructions. Relative cell growth was determined as a percentage of untreated control cells.

### Transwell migration and invasion assay

A total of 2 × 10^5^ cells were seeded on transwell chambers, and treated with normal monocytes medium or medium from CAFs‐stimulated monocytes. Then the cells were allowed to migrate through the filter for 24 h. The migrated cells were identified using crystal violet. The number of cells in five 100× fields was counted in each group. For invasion assay, transwell chambers were pre‐coated with gel matrix (Matrigel; BD Franklin Lakes, NJ),

### Statistical analysis

Statistical analysis was performed by GraphPad Prism 5.0 software (La Jolla, CA). All data were represented as the mean with error bars corresponding to SEM. Differences between groups were compared using unpaired two‐tailed Student's t test or ANOVA with Bonferroni's multiple comparison tests. *P *<* *0.05 was considered statistically significant.

## Results

### Characterization of CAFs from human PDAC

**Figure 1:**
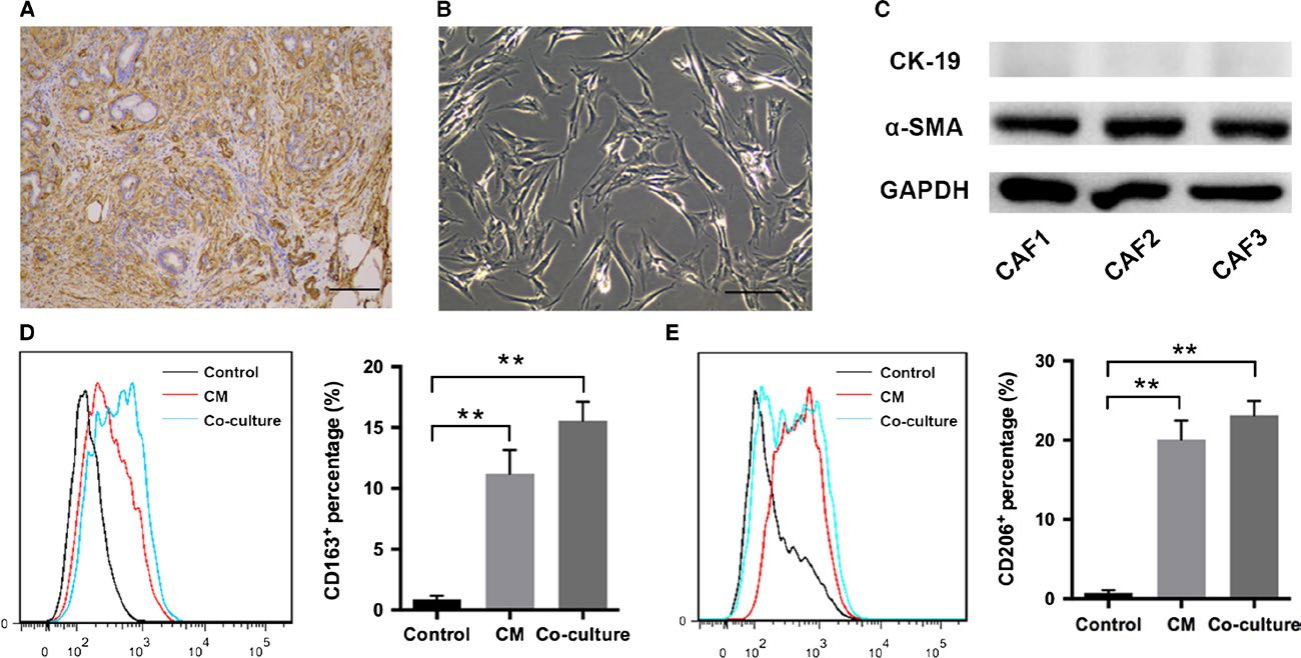
The characterization of cancer‐associated fibroblasts (CAFs) isolated from human pancreatic ductal adenocarcinoma (PDAC). Immunohistochemistry showed abundant *α*‐SMA ^+^ CAFs from a PDAC sample. The scale bar is 100 *μ*m (A). The primary CAFs exhibited typical spindle‐like mesenchymal morphology. The scale bar is 20 *μ*m (B). The expression for *α*‐SMA in CAFs was analyzed by western blot (C). The polarization status for human monocytes either treated by CAFs CM or directly co‐cultured with CAFs was detected by flow cytometry analysis for CD206 or CD163 expression (D–E). ** indicated *P *<* *0.01.

Pancreatic CAFs were generated from resected specimens in three PDAC patients undergoing curative surgery. *α*‐SMA was widely used as a marker for activated CAFs, and immunohistochemical examination showed that *α*‐SMA^+^ CAFs were abundant in the stromal compartment from the PDAC tissue (Fig. 1A). The isolated primary pancreatic CAFs exhibited typical features of spindle‐like mesenchymal cells (Fig. 1B). Western blot analysis further confirmed the phenotype of CAFs that are positive for *α*‐SMA and negative for epithelial marker CK19 (Fig. 1C).

### Pancreatic CAFs led to M2 polarization of monocytes

To investigate the effect of CAFs on the polarization states of monocytes, CD14^+^ human PBMC were treated with CM from pancreatic CAFs. After stimulation for 48 h, a significant increased monocytes population showed expression of CD206 compared with nontreated cells (*P *<* *0.01; Fig. 1D). The staining for another M2 marker, CD163 also exhibited enhanced expression upon CM stimulation (*P *<* *0.01; Fig. 1E). Then, a co‐culture system for CAFs and PBMC was made and similarly, the percentage of M2 phenotype monocytes was markedly elevated in contrast to the control group (*P *<* *0.01; Fig. 1D–E). Interestingly, we noticed that the percentage of CD206^+^ or CD163^+^ monocytes in co‐culture group tend to be higher than those in CM group, though the difference was not significant (Fig. 1D–E).

### The M2 phenotype transformation induced by pancreatic CAFs was caused by increased ROS production in monocytes

**Figure 2:**
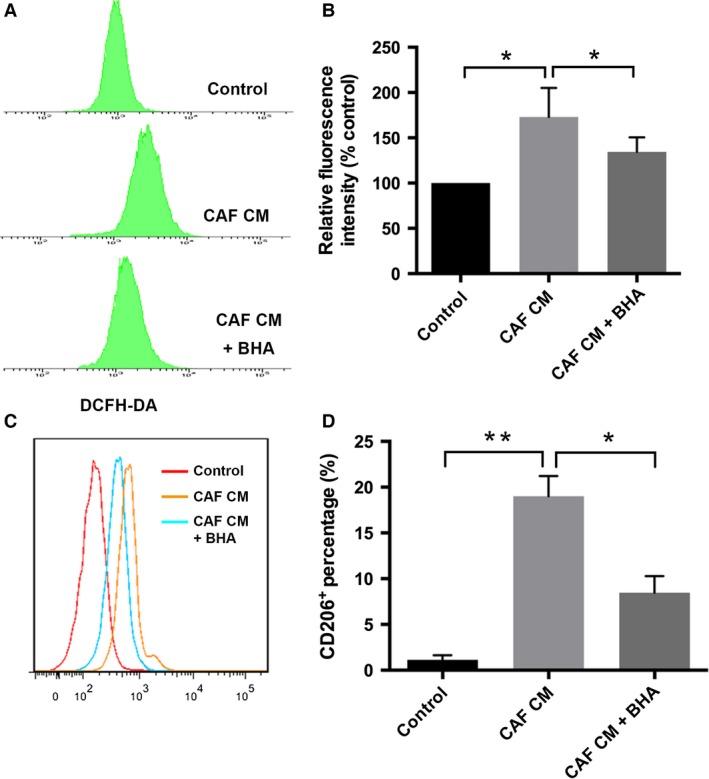
The M2 phenotype induced by pancreatic CAFs was caused by increased reactive oxygen species (ROS) production. Monocytes were treated by CAFs CM with or without antioxidant BHA, and ROS level was measured through DCFH‐DA (A–B). M2 polarization was examined via flow cytometry analysis of CD206 expression (C–D). ** indicated *P *<* *0.01, * indicated *P *<* *0.05.

It has been shown that oxidative stress played a role in modulating macrophage phenotype, and thus ROS status was evaluated next. When monocytes were stimulated by CAFs CM, ROS production was remarkably elevated (*P *<* *0.05; Fig. 2A–B) and the percentage of CD206^**+**^ monocytes also increased (*P *<* *0.01; Fig. 2C–D). But upon elimination of ROS by BHA (*P *<* *0.05; Fig. 2A–B), the effect on M2 polarization was significantly abolished, suggesting a critical role of ROS in CAF‐induced phenotype change (*P *<* *0.05; Fig. 2C–D).

### Secreted M‐CSF from pancreatic CAF led to enhanced ROS production and M2 polarization in monocytes

**Figure 3:**
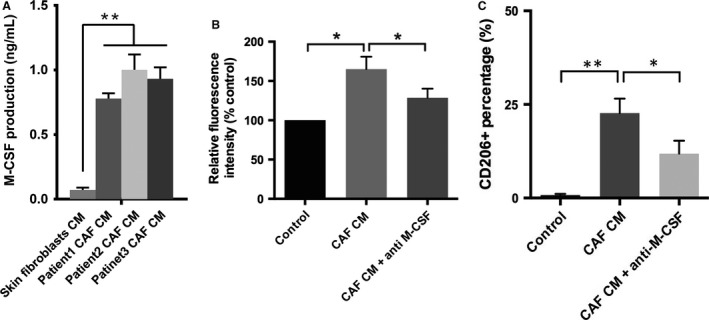
Secreted M‐CSF from pancreatic CAFs led to enhanced reactive oxygen species (ROS) production and M2 polarization. The secretion of soluble factors by CAFs was determined by ELISA (A). ROS level was measured when M‐CSF was blocked (B). The effect of blocking M‐CSF on the M2 polarization was evaluated by flow cytometry (C). * indicated *P *<* *0.05, ** indicated *P *<* *0.01.

To further delineate the mechanism of CAFs‐induced phenotype change in monocytes, we sought to determine the soluble factors in CAFs supernatant. In particular, the secretion of M‐CSF from pancreatic CAFs was abundant compared with skin fibroblasts (Fig. 3A). M‐CSF has been suggested to be associated with macrophage differentiation and is consistently increased in all three pancreatic CAF cell lines here. To corroborate the role of M‐CSF, a specific M‐CSF neutralizing antibody was used to block its effects. The DCFH‐DA test showed that M‐CSF blockade significantly decreased ROS production (*P *<* *0.05; Fig. 3B). When M‐CSF was inhibited, the induction of M2 polarization by CAF CM was also partly abrogated (*P *<* *0.05; Fig. 3C). These data indicated that the M‐CSF from CAFs contribute to the M2 polarization through increased generation of ROS in monocytes.

### CAFs‐stimulated macrophages exhibited pro‐tumor effect on pancreatic tumor cell lines

**Figure 4:**
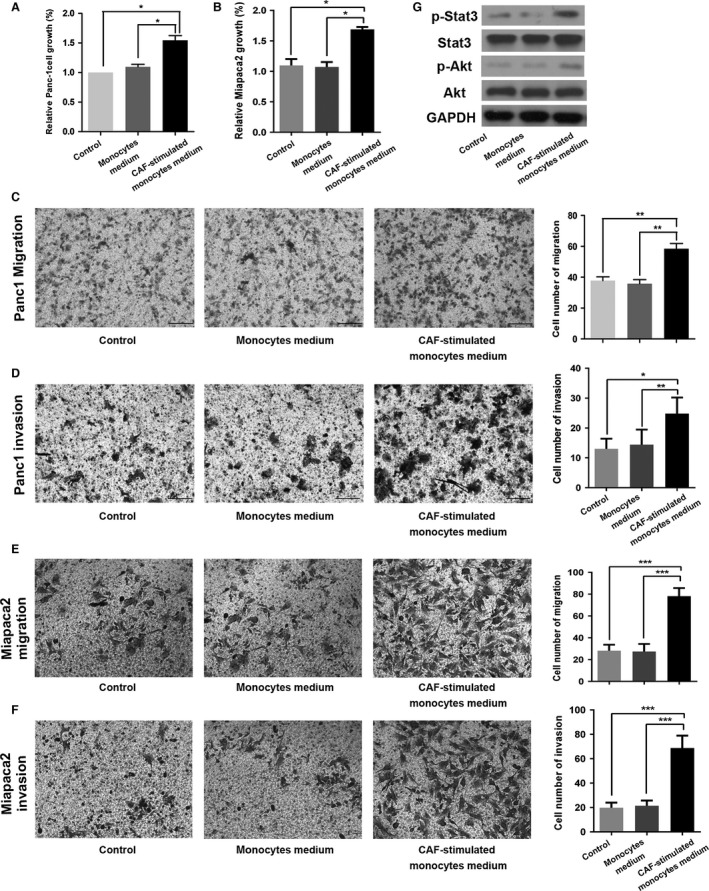
The effect of CAFs‐induced M2 macrophages on pancreatic cancer cells. The effect of CAFs‐induced M2 phenotype on Panc1 and Miapaca2 cells growth (A–B), migration (C–D), and invasion (E–F). Western blot analysis for p‐Stat3 and p‐Akt (Fig. 4G). * indicated *P *<* *0.05, ** indicated *P *<* *0.01.

We examined the impact of CAFs‐stimulated M2 macrophage on pancreatic cancer cells. The proliferation assay showed that M2 macrophage significantly enhanced Panc1 and Miapaca2 cell growth compared with control or normal monocytes‐stimulated group (*P *<* *0.05; Fig. 4A–B). The transwell assay demonstrated that the induced M2 macrophage substantially enhanced the migration and invasion of Panc1 and Miapaca2 cells (Fig. 4C–F). We further explored the mechanisms of enhanced tumor progression. Panc1 cells exhibit increased p‐Stat3 and p‐Akt expression when treated with conditioned medium from CAFs‐stimulated macrophages (Fig. 4G), indicating that Stat3/Akt pathway might contribute to the enhanced tumor progression.

## Discussion

CAFs and TAM are major contributors to the stromal evolution in PDAC, but their interaction remains largely unknown. In this study, our data show that pancreatic CAFs play an important role in regulating macrophage phenotype. CAFs are able to induce M2 polarization partly through paracrine secretion of M‐CSF, in that blockade of M‐CSF signaling markedly attenuates the generation of M2 macrophages. In addition, CAFs can enhance pancreatic cancer growth and progression via inducing M2 reprogramming of macrophages. Therefore, we suggest that the interaction between CAFs and macrophages might be a novel target in ameliorating the pro‐tumorigenic microenvironment and treatment of PDAC.

The generation of M2 phenotype macrophage has long been believed to be due to the interaction with tumor cells. However, in the milieu of PDAC, activated myofibrolasts dominates over tumor cells and is implied to play a critical role in modulating immune microenvironment. Previous studies have confirmed that CAFs could direct tumor immune evasion by secreting CXCL12 in a mouse model of human PDAC 8. There are also evidence that pancreatic CAFs are associated with recruitment and differentiation of myeloid‐derived suppressive cells 13. When CAFs were depleted in a spontaneous pancreatic cancer model, dramatic change took place, in particular the tumor immune microenvironment involving different kinds of T cells, myeloid cells, and macrophages as well 14. Our results further confirmed that CAFs also have a role in modulating M2 reprogramming, through which it might facilitate the immune suppression. As a result, it is speculated that CAFs might have a broad effect in the recruitment and phenotype regulation of immune cell.

Among the secreted cytokines by pancreatic CAFs, we found abundant M‐CSF, which is consistent with previous study 13. M‐CSF has been shown to exert its effects on macrophages via CSF1R and polarize TAM into tumor‐promoting phenotypes 15. In fact, Zhu Y et al. provided evidence that PDAC tumor cells overexpressed M‐CSF, which resulted in the activation of CSF1R in macrophages and subsequent M2 transformation 16. Our data further suggest CAFs could also be a source of M‐CSF within PDAC milieu.

In addition, this study implicated ROS as being a crucial component in M2 phenotype modulation, which is consistent with previous report 17. Indeed, the role of ROS in cancer‐like PDAC has been intensely studied but was mainly examined within tumor cells. The evidence presented conflicting results of either pro‐ or anti‐tumor effect regarding tumor cell intrinsic ROS 18. In contrast, our data showed that ROS can also affect tumor progression by targeting macrophages, further complicating the function of ROS in PDAC. Thus, the treatment strategy by inhibiting ROS generation requires further studies.

CAFs in PDAC have long been seen as a malicious component and promote cancer proliferation, invasion, and metastasis in a paracrine fashion. This study also supports its tumor‐promoting effect. In a preclinical study, Oliver et al. demonstrated that hedgehog inhibitor decreased the number of CAFs and thereafter exerted significant anti‐tumor effect either alone or combined with gemcitabine 19. However, the adoption of cyclopamine, a specific Hh antagonism, failed to show efficacy in a few clinical trials. Recent findings suggested that CAFs had a paradoxical restraining effect on pancreatic tumors. There are two studies providing evidence that inhibition CAFs proliferation via long‐term blockade of hedgehog signaling accelerated tumor growth and progression 20, 21. Therefore, the available evidence might imply a complex role of CAFs in PDAC acting as either pro‐ or anti‐tumor factor through distinct mechanisms.

In conclusion, this study examined the relationship between CAFs and TAM in PDAC, and indicated CAFs as a source of soluble cytokines modulating the phenotype of other stromal cells. Studies should be performed to further decipher the complicated function of CAFs in PDAC.

## Conflict of Interest

No conflict of interest exists.
